# Predictive value of circulating PD-1^hi^CXCR5^−^ peripheral T helper cells in systemic inflammatory response syndrome after percutaneous nephrolithotomy

**DOI:** 10.3389/fmed.2025.1483273

**Published:** 2025-03-12

**Authors:** Zhenyu Liu, Wei Zhu, Wentao Yu, Yu Zhou, Xiaojing Dai, Yan Wang, Jingxuan Yu, Lin Wang, Yanbin Niu, Ling Yang, Sen Xie, Ping Long, Guohua Zeng, Lei Gao, Tiejun Pan

**Affiliations:** ^1^Department of Urology, General Hospital of Central Theater Command of Chinese People's Liberation Army, Wuhan, Hubei, China; ^2^Department of Urology and Guangdong Key Laboratory of Urology, The First Affiliated Hospital of Guangzhou Medical University, Guangzhou, Guangdong, China; ^3^Postdoctoral Research Workstation, General Hospital of Central Theater Command of Chinese People's Liberation Army, Wuhan, Hubei, China; ^4^Department of Urology, The 985th Hospital of the Chinese People's Liberation Army Joint Logistic Support Force, Taiyuan, Shanxi, China

**Keywords:** peripheral T helper cell (Tph), kidney stone, nephrolithiasis, percutaneous nephrolithotomy, systemic inflammatory response syndrome

## Abstract

**Purpose:**

The principal objective of this study was to investigate the potential risk factors contributing to the development of postoperative systemic inflammatory response syndrome (SIRS) after percutaneous nephrolithotomy (PCNL), with a key focus on a novel subpopulation of PD-1^hi^CXCR5^−^CD4^+^ T cells, termed peripheral T helper (Tph) cells.

**Methods:**

A comprehensive retrospective analysis was undertaken on 399 patients with kidney stones who underwent PCNL at two hospitals between January 2022 and December 2023. The core outcome of interest was the occurrence of post-PCNL SIRS. Univariate and multivariate logistic regression analysis were performed to elucidate independent risk factors for post-PCNL SIRS. A precise nomogram was constructed, integrating the independent risk factors, including Tph cell levels, and receiver operating characteristic (ROC) curves and calibration curves were generated.

**Results:**

Among the patients, 142 (35.59%) developed post-PCNL SIRS. Univariate analysis highlighted eight potential risk factors. Notably, multivariate analysis identified five independent risk factors for post-PCNL SIRS: high stone density (odds ratio [OR], 5.96; *p* < 0.001), prolonged operation time (OR, 2.26; *p* = 0.005), absence of hydronephrosis (OR, 0.37; *p* < 0.001), positive urine detection for bacteria (OR, 2.13; *p* = 0.003) and low percentage of circulating Tph cells (OR, 0.39; *p* < 0.001).

**Conclusion:**

Patients presenting with low circulating Tph cell levels, high stone density, prolonged operation time, absence of hydronephrosis, and positive urine bacteria are at an elevated risk of developing post-PCNL SIRS. For these individuals, careful consideration of preoperative evaluations, heightened vigilance, and appropriate treatment strategies are essential.

## Introduction

Nephrolithiasis is a prevalent and recurrent urological disorder, with a global incidence spanning 1% to 13% (1, 2). Over half of the afflicted individuals experience relapse within a decade, frequently culminating in colic, infection, compromised renal function, and occasionally, renal failure. The therapeutic landscape for nephrolithiasis has transitioned from traditional open surgery to minimally invasive endoluminal urological interventions, with percutaneous nephrolithotomy (PCNL) emerging as the foremost treatment modality for patients harboring staghorn calculi, complex stones, and most upper urinary tract stones exceeding 20 mm in diameter. PCNL boasts advantages over traditional open surgery, including minimal invasiveness, expedited hospital discharge, shortened operative durations, and heightened stone clearance rates.

Nonetheless, the intricate nature of PCNL procedures and its protracted learning curve contribute to a heightened postoperative complication rate, notably postoperative infection and hemorrhage, with sepsis standing as a particularly pernicious and prevalent complication. Sepsis is defined as a life-threatening condition characterized by organ dysfunction stemming from a dysregulated host response to infection (3). Systemic inflammatory response syndrome (SIRS) is a critical complication following PCNL, with reported incidence rates ranging from 22.1% to 43.0% in various studies (3). SIRS not only increases the risk of progression to sepsis, a life-threatening condition characterized by organ dysfunction, but also significantly prolongs hospital stays, escalates healthcare costs, and adversely affects patient outcomes (4–6). The development of post-PCNL SIRS is associated with a higher likelihood of requiring intensive care, extended antibiotic therapy, and additional interventions, which can further strain healthcare resources. Moreover, patients who develop SIRS are at an increased risk of long-term renal impairment and other systemic complications, underscoring the importance of early identification and intervention (6). Despite advances in surgical techniques and perioperative care, the incidence of post-PCNL SIRS remains substantial, highlighting the need for reliable preoperative biomarkers to identify high-risk patients and implement targeted preventive strategies.

Recent research endeavors have implicated C-reactive protein, stone culture, stone burden, and operative duration as potential risk factors for post-PCNL SIRS (7). However, their predictive accuracy has proven inconsistent across various studies. Moreover, certain indicators, such as stone composition analysis and stone culture, present preoperative accessibility challenges. Therefore, the identification of efficacious preoperative biomarkers capable of accurately predicting post-PCNL SIRS holds paramount significance.

Peripheral T helper (Tph) cells, a newly delineated subset of CD4^+^ T cells, have garnered attention for their pathogenic role in rheumatoid arthritis (RA) (8). These cells, initially identified by mass cytometry in RA joint tissue and distinguished by their PD-1^hi^CXCR5^−^ phenotype, exhibit potent B-cell-helper function. Tph cells sorted from peripheral blood, synovial fluid and synovial tissue alike have been shown to induce co-cultured memory B cell differentiation into plasma cells and augment IgG production *in vitro*, suggesting a role in combating bacterial and viral infections (9). This B-cell-helper function is hypothesized to be mediated by cytokine IL-21, as evidenced by inhibited plasma cell differentiation upon IL-21 neutralization in co-culture systems (8, 10). Notably, increased circulating PD-1^hi^CXCR5^−^CD4^+^ Tph cells have been observed in patients with IgG4-related disease (11), systemic lupus erythematosus (10), type 1 diabetes (12), IgA nephropathy (13), psoriasis vulgaris (14) and active ulcerative colitis (15), further underscoring their significance.

Given the robust B-cell-helper function of Tph cells, their potential as predictive biomarker for post-PCNL SIRS is intriguing. However, to date, no study has examined the association between this Tph subset and post-PCNL SIRS. Consequently, the present investigation retrospectively analyzed the feasibility of utilizing Tph cells as a biomarker for post-PCNL SIRS.

## Materials and methods

### Study population

The retrospective analysis in this study encompassed all adult patients who underwent PCNL at the General Hospital of Central Theater Command and the First Affiliated Hospital of Guangzhou Medical University, spanning from January 2022 and December 2023. Eligibility criteria for inclusion were: (1) attainment of an age threshold of 18 years or older, (2) undergoing PCNL as the primary surgical intervention, and (3) availability of preoperative hematological parameters and computed tomography (CT) imaging. Conversely, exclusion criteria were devised to exclude: (1) patients with a prior or current diagnosis of malignancy, (2) those suffering from hematopathy, immune dysfunction, or undergoing/having undergone immune-modulatory therapies, (3) the presence of active infectious diseases or fever at the time of intervention, and (4) recent use of antibiotics within 1 month prior to blood sampling. This study adhered rigorously to the principles outlined in the Declaration of Helsinki and received ethical clearance from the Medical Ethical Committee of the General Hospital of Central Theater Command (No: 2023-016-01). Furthermore, all participants provided written informed consent, in accordance with the mandates of the Declaration of Helsinki.

### Data collection

Routine blood and urine analysis, as well as biochemical evaluations, were performed prior to PCNL. Lymphocyte subsets were meticulously assessed using flow cytometry. Peripheral blood mononuclear cells (PBMCs) were isolated from heparinized peripheral venous blood obtained from nephrolithiasis patients prior to PCNL, employing density gradient centrifugation with Lymphprep^TM^ (STEMCELL). For analyzing Tph and follicular helper T (Tfh) cells, the isolated PBMCs were labeled with a panel of antibodies: anti-CD3-PE/Cy7 (HIT3a; Biolegend), anti-CD4-PerCP/Cy5.5 (OKT4; Biolegend), anti-CD45RO-FITC (UCHL1; Biolegend), anti-PD1-APC (EH12.2H7; Biolegend), anti-CXCR5 (J252D4; Biolegend) and LIVE/DEAD fixable near-IR dead cell dye (Thermofisher). Sample acquisition was conducted on a FACSCanto II flow cytometer (BD Biosciences, San Jose, CA, USA), data were analyzed using FlowJo software version 10. Additionally, all patients underwent CT to confirm stone characteristics. The stone burden was estimated using Ackermann's formula (16).

### Surgical technique and treatment strategy

Patients with nephrolithiasis were managed according to the most recent guidelines issued by the European Association of Urology (17). For patients with a negative uring culture, 1 g of ceftriaxone was administered intravenously 1 h prior to anesthetic induction. Conversely, patients with a positive urine culture received antibiotics tailored to the antibiotic susceptibility map, commencing 5 days before surgery and reiterated 1 h before anesthesia (18). All surgical procedures were executed by two experienced surgeons proficient in PCNL, working across two hospitals. A brief overview of the surgical process is as follows: under general anesthesia, a ureteral catheter was inserted into the ureter of the patient in the lithotomy position via ureteroscopy. Subsequently, the patient was positioned prone, and the optimal percutaneous access was established by puncturing the appropriate renal calyx under ultrasound guidance. This was followed by dilation of the percutaneous tract using a fascial dilator to accommodate an 18-Fr sheath. A perfusion flow rate of approximately 400 ml/min was maintained, and stone fragmentation was achieved using either a pneumatic device or a holmium laser. postoperatively, a 6-Fr double-J stent and a 16-Fr nephrostomy tube were routinely placed in accordance with standard protocols. Operation time was defined as the interval from renal calyx puncture to nephrostomy tube placement. On the second postoperative day, the nephrostomy tube was clamped and, if tolerated (i.e., mild or no pain), removed after 24 h. The double-J stent was removed cystoscopically four weeks after surgery. Follow-up evaluations, conducted 2–3 months postoperatively, encompassed assessment of stone-free status, routine urine analysis, and renal function. Patients requiring a second-stage procedure underwent this intervention 2 weeks or more post-discharge, as necessary.

### Endpoints

The primary endpoint of this study was the development of post-PCNL SIRS during the postoperative hospitalization period. All patients underwent routine monitoring until discharge. Post-PCNL SIRS was deemed to have occurred if two or more of the subsequent criteria were fulfilled: (1) heart rate exceeding 90 bpm; (2) body temperature above 38°C or below 36°C; (3) respiratory rate surpassing 20 breaths per min; (4) white blood cell counts either exceeding 12 × 10^9^ cells/L or falling below 4 × 10^9^ cells/L.

### Statistical analysis

Statistical analyses were conducted using R software (version 4.3.3). Data were reported in the form of medians, quartiles, or percentages as applicable. Continuous variables adhering to the normal distribution were evaluated using independent t-sample tests, whereas non-parametric tests were applied to those continuous variables that did not conform to the normal distribution (19). Categorical variables were analyzed with the chi-square test. To identify independent risk factors for post-PCNL SIRS, variables with *p* < 0.05 in the univariate analysis were subsequently included in the multivariate analyses, which were performed through backward stepwise selection. Odds ratios (ORs) and 95% confidence intervals (CIs) were computed. All *p*-values were two-sided, and a threshold of < 0.05 was considered statistically significant. Spearman's correlation test was used to analyze correlations between Tph cells and clinical outcomes.

## Results

In this study, a cohort of 399 patients who underwent one-stage PCNL for kidney and upper urinary tract stones were ultimately included. Among these patients, 58.65% (*n* = 234) were male, with the remaining 41.35% (n=165) being female. Notably, 142 patients (35.59%) developed post-PCNL SIRS. The characteristics of the post-PCNL SIRS group, compared to the non-SIRS group, were marked by elevated stone density (*p* < 0.001), prolonged operative duration (*p* = 0.002), negative hydronephrosis (*p* < 0.001), increased white blood cell (WBC) counts (*p* = 0.039), low serum albumin levels (*p* = 0.030), positive urine leukocytes (*p* = 0.043), positive urine detection for bacteria (*p* < 0.001), and decreased percentage of Tph cell (*p* < 0.001) (Tables 1, 2). However, no significant difference was observed in percentage of follicular helper T (Tfh) cell between the post-PCNL SIRS and non-SIRS groups (Table 2).

**Table 1:** Basic characteristics data in the non-SIRS group and the SIRS group.

**Variables**	**Non-SIRS (*n* = 257)**	**SIRS (*n* = 142)**	***p* value**
**Demographics**			
Age, median (IQR) (years)	58.00 (49.00–65.00)	55.00 (43.00–61.00)	0.051
Male, gender, *n* (%)	158 (61.48%)	76 (53.52%)	0.150
BMI, median (IQR)	24.03 (21.83–26.03)	23.86 (21.38–25.64)	0.116
**Clinical characteristics**			
Diabetes, *n* (%)	45 (17.51%)	24 (16.90%)	0.988
Hypertension, *n* (%)	84 (32.68%)	39 (27.46%)	0.333
Stone density, median (IQR) (HU)	1,185 (784–1,505)	1,482 (1,155–1,743)	<0.001^*^
Operation time, median (IQR) (min)	105 (73–150)	112 (85–163)	0.002^*^
Stone burden, median (IQR) (mm^2^)	612.00 (312.00–1,410.00)	519.38 (270.01–978.00)	0.404
Staghorn calculi, *n* (%)	80 (31.13%)	47 (33.10%)	0.770
**Stone side**, ***n*** **(%)**			0.496
Left	127 (49.42%)	76 (53.52%)	
Right	130 (50.58%)	66 (46.48%)	
**Hydronephrosis**, ***n*** **(%)**			<0.001^*^
Yes	201 (78.21%)	77 (54.23%)	
No	56 (21.79%)	65 (45.77%)	
**Routine blood tests**			
WBC, median (IQR) (109/L)	6.69 (5.60–8.05)	6.80 (5.80–8.60)	0.039^*^
Lymphocytes, median (IQR) (109/L)	1.90 (1.50–2.24)	1.94 (1.40–2.34)	0.592
Neutrophils, median (IQR) (109/L)	4.00 (3.09–5.01)	4.05 (3.10–5.76)	0.603
Hemoglobin, median (IQR) (g/L)	134.00 (122.00–146.00)	132.00 (117.00–144.00)	0.165
Total protein, median (IQR) (g/L)	70.40 (65.60–74.20)	69.70 (65.70–73.90)	0.955
Albumin, median (IQR) (g/L)	41.20 (38.20–43.10)	40.20 (37.70–42.40)	0.030^*^
Globulin, median (IQR) (g/L)	24.50 (20.10–28.30)	25.30 (20.00–29.30)	0.950
Albumin-globulin ratio	1.65 (1.42–2.01)	1.58 (1.36–2.05)	0.469
**Kidney function**			
Creatinine, median (IQR) (μmol/L)	82.00 (67.90–104.30)	76.40 (63.80–95.30)	0.242
BUN, median (IQR) (mol/L)	5.86 (4.95–7.12)	5.51 (4.60–6.38)	0.136
Uric acid, median (IQR) (μmol/L)	378.80 (319.75–456.28)	365.00 (296.00–431.20)	0.111
**Urine examination**			
**Hematuria**, ***n*** **(%)**			0.525
Positive	217 (84.44%)	124 (87.32%)	
Negative	40 (15.56%)	18 (12.68%)	
**Urine leukocytes**, ***n*** **(%)**			0.043^*^
Positive	181 (70.43%)	114 (80.28%)	
Negative	76 (29.57%)	28 (19.72%)	
**Urine protein**, ***n*** **(%)**			0.999
Positive	69 (26.85%)	38 (26.76%)	
Negative	188 (73.15%)	104 (73.24%)	
**Urine nitrite**, ***n*** **(%)**			0.005
Positive	24 (9.34%)	28 (19.72%)	
Negative	233 (90.66%)	114 (80.28%)	
**Urine bacteria**, ***n*** **(%)**			<0.001^*^
Positive	69 (26.85%)	67 (47.18%)	
Negative	188 (73.15%)	75 (52.82%)	

^*^p < 0.05; WBC, white blood cells; BUN, blood urea nitrogen.

**Table 2:** Cytokine and lymphocyte subsets in the SIRS and the non-SIRS group.

**Variables**	**Non-SIRS (*n* = 257)**	**SIRS (*n* = 142)**	***p* value**
IL-6, median (IQR), pg/mL	24.05 (11.38–84.35)	43.00 (12.10–196.55)	0.064
CD4+ T cells, median (IQR), %	42.00 (22.00–60.90)	45.95 (27.50–63.90)	0.639
CD4+CD45RO+ T cells, median (IQR), %	22.40 (11.50–33.60)	24.80 (13.50–36.38)	0.584
CD4+CD45RO+CXCR5+ PD-1+ Tfh cells, median (IQR), %	7.60 (4.20–10.70)	6.85 (3.78–10.60)	0.531
CD4+CD45RO+CXCR5-PD-1+ Tph cells, median (IQR), %	20.60 (12.50–24.80)	10.80 (7.00–15.88)	<0.001^*^

^*^p < 0.05; IL-6, interleukin 6; Tfh cells, follicular helper T cells; Tph cells, peripheral T helper cells.

**Figure 1:**
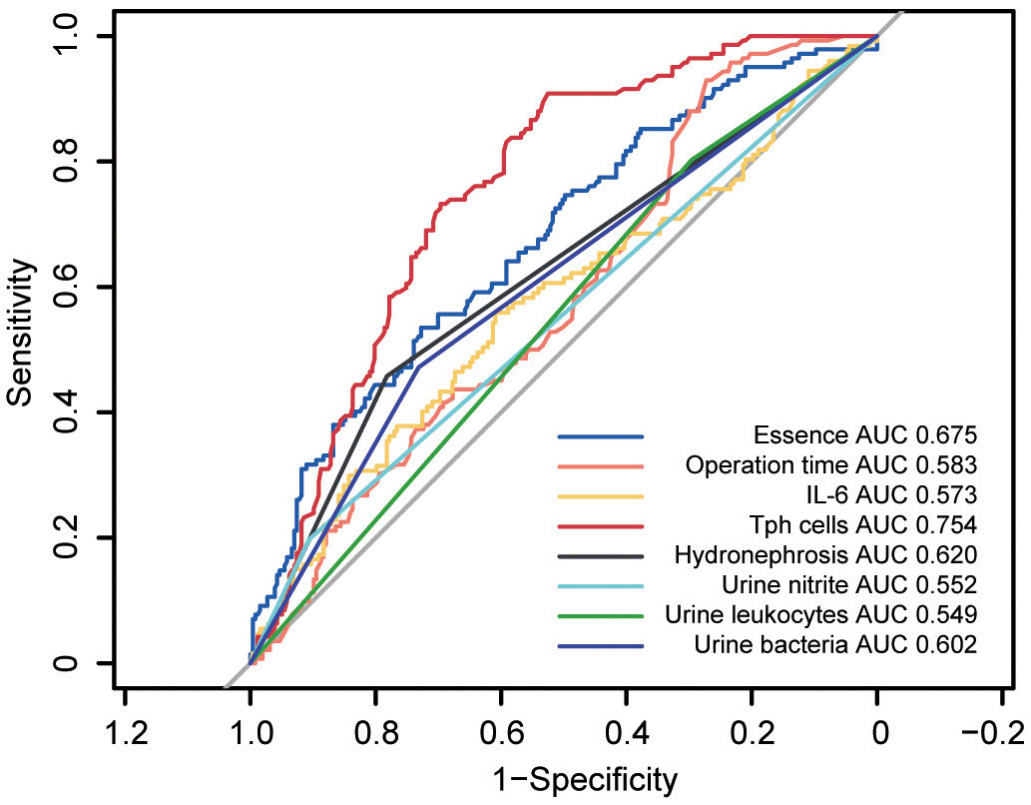
Receiver operating characteristics (ROC) curves for univariate analyses of potential risk factors in post-PCNL SIRS. ROC curves for univariate analyses of potential risk factors in post-PCNL SIRS showed the area under curve (AUC) for circulating Tph cells was 0.754, highest among other potential risk factors.

To identify potential risk factors for post-PCNL SIRS, a univariate analysis was performed on all included variables. Eight factors emerged as potential risk factors: stone density (*p* < 0.001, OR = 5.15, 95%CI = 2.79–9.50), operation time (*p* < 0.001, OR = 2.16, 95%CI = 1.33–3.51), hydronephrosis (*p* < 0.001, OR = 0.33, 95%CI = 0.21–0.51), urine nitrite positivity (*p* < 0.001, OR = 2.38, 95%CI = 1.32–4.30), positive urine leukocytes (*p* = 0.030, OR = 1.71, 95%CI = 1.04–2.80), positive urine detection for bacteria (*p* < 0.001, OR = 2.43, 95%CI = 1.58–3.74), IL-6 levels (*p* = 0.020, OR = 1.15, 95%CI = 1.03–1.29) and percentage of Tph cell (*p* < 0.001, OR = 0.34, 95%CI = 0.25–0.47) (Table 3). Among these, receiver operating characteristic (ROC) curve analysis revealed that Tph cells had the highest area under the curve (AUC) of 0.754 (Figure 1).

**Table 3:** Univariate analyses of risk factors for SIRS.

**Variables**	**OR (95% CI)**	***p* value**
Stone density	5.15 (2.79–9.50)	<0.001^*^
Operation time	2.16 (1.33–3.51)	<0.001^*^
Hydronephrosis	0.33 (0.21–0.51)	<0.001^*^
Urine nitrite	2.38 (1.32–4.30)	<0.001^*^
Urine leukocytes	1.71 (1.04–2.80)	0.030^*^
Urine bacteria	2.43 (1.58–3.74)	<0.001^*^
IL-6	1.15 (1.03–1.29)	0.020^*^
Percentage of CD4+ T cells	1.11 (0.84–1.47)	0.450
Percentage of CD4+CD45RO+ T cells	1.17 (0.88–1.54)	0.280
Percentage of CD4+CD45RO+CXCR5-PD-1+ Tph cells	0.34 (0.25–0.47)	<0.001^*^
Percentage of CD4+CD45RO+CXCR5+PD-1+ Tfh cells	0.89 (0.67–1.20)	0.450

^*^p < 0.05; IL-6, interleukin 6; Tfh cells, follicular helper T cells; Tph cells, peripheral T helper cells.

To ascertain independent risk factors for post-PCNL SIRS, a multivariate regression analysis was conducted, incorporating the eight risk factors identified in the univariate analysis. Through backward stepwise selection, four models with five independent risk factors each were established. Model 1 included stone density (*p* < 0.001, OR = 5.90, 95%CI = 3.00–11.62), operation time (*p* = 0.007, OR=2.18, 95%CI = 1.24–3.84), hydronephrosis (*p* < 0.001, OR = 0.35, 95%CI = 0.21–0.58), urine nitrite positivity (*p* = 0.037, OR = 2.06, 95%CI = 1.04–4.08) and the percentage of Tph cell (*p* < 0.001, OR = 0.38, 95%CI = 0.27–0.55) (Table 4). Similarly, Model 2 featured stone density (*p* < 0.001, OR = 6.12, 95%CI = 3.11–12.07), operation time (*p* = 0.004, OR = 2.18, 95%CI = 1.29–4.01), hydronephrosis (*p* < 0.001, OR = 0.35, 95%CI = 0.21–0.58), urine leukocytes (*p* = 0.025, OR = 1.91, 95%CI = 1.08–3.38), and the percentage of Tph cell (*p* < 0.001, OR = 0.38, 95%CI = 0.26–0.54) (Table 4). Model 3 comprised stone density (*p* < 0.001, OR = 5.96, 95%CI = 3.01–11.81), operation time (*p* = 0.005, OR = 2.26, 95%CI = 1.28–4.00), hydronephrosis (*p* < 0.001, OR = 0.37, 95%CI = 0.22–0.62), urine detection for bacteria (*p* = 0.003, OR = 2.13, 95%CI = 1.30–3.48), and the percentage of Tph cell (*p* < 0.001, OR = 0.39, 95%CI = 0.27–0.55) (Table 4). Lastly, Model 4 included stone density (*p* < 0.001, OR = 5.63, 95%CI = 2.87–11.05), hydronephrosis (*p* < 0.001, OR = 0.35, 95%CI = 0.21–0.58), urine nitrite (*p* = 0.044, OR = 2.04, 95%CI = 1.02–4.07), urine leukocytes (*p* = 0.049, OR = 1.77, 95%CI = 1.00–3.13), and the percentage of Tph cell (*p* < 0.001, OR = 0.37, 95%CI = 0.26–0.52) (Table 4). Notably, stone density, hydronephrosis and the percentage of Tph cell were consistently present in all four multivariate regression models.

**Table 4:** Multivariate analyses of risk factors for SIRS.

**Variables**	**Model 1**		**Model 2**		**Model 3**		**Model 4**	
	**OR (95% CI)**	***p* value**	**OR (95% CI)**	***p* value**	**OR (95% CI)**	***p* value**	**OR (95% CI)**	***p* value**
Stone density	5.90 (3.00–11.62)	<0.001	6.12 (3.11–12.07)	<0.001	5.96 (3.01–11.81)	<0.001	5.63 (2.87–11.05)	<0.001
Operation time	2.18 (1.24–3.84)	0.007	2.28 (1.29–4.01)	0.004	2.26 (1.28–4.00)	0.005	–	–
Hydronephrosis	0.35 (0.21–0.58)	<0.001	0.35 (0.21–0.58)	<0.001	0.37 (0.22–0.62)	<0.001	0.35 (0.21–0.58)	<0.001
Urine nitrite	2.06 (1.04–4.08)	0.037	–	–	–	–	2.04 (1.02–4.07)	0.044
Urine leukocytes	–	–	1.91 (1.08–3.38)	0.025	–	–	1.77 (1.00–3.13)	0.049
Urine bacteria	–	–	–	–	2.13 (1.30–3.48)	0.003	–	–
Percentage of CD4+CD45RO+CXCR5-PD-1+ Tph cells	0.38 (0.27–0.55)	<0.001	0.38 (0.26–0.54)	<0.001	0.39 (0.27–0.55)	<0.001	0.37 (0.26–0.52)	<0.001

Tph cells, peripheral T helper cells.

**Figure 2:**
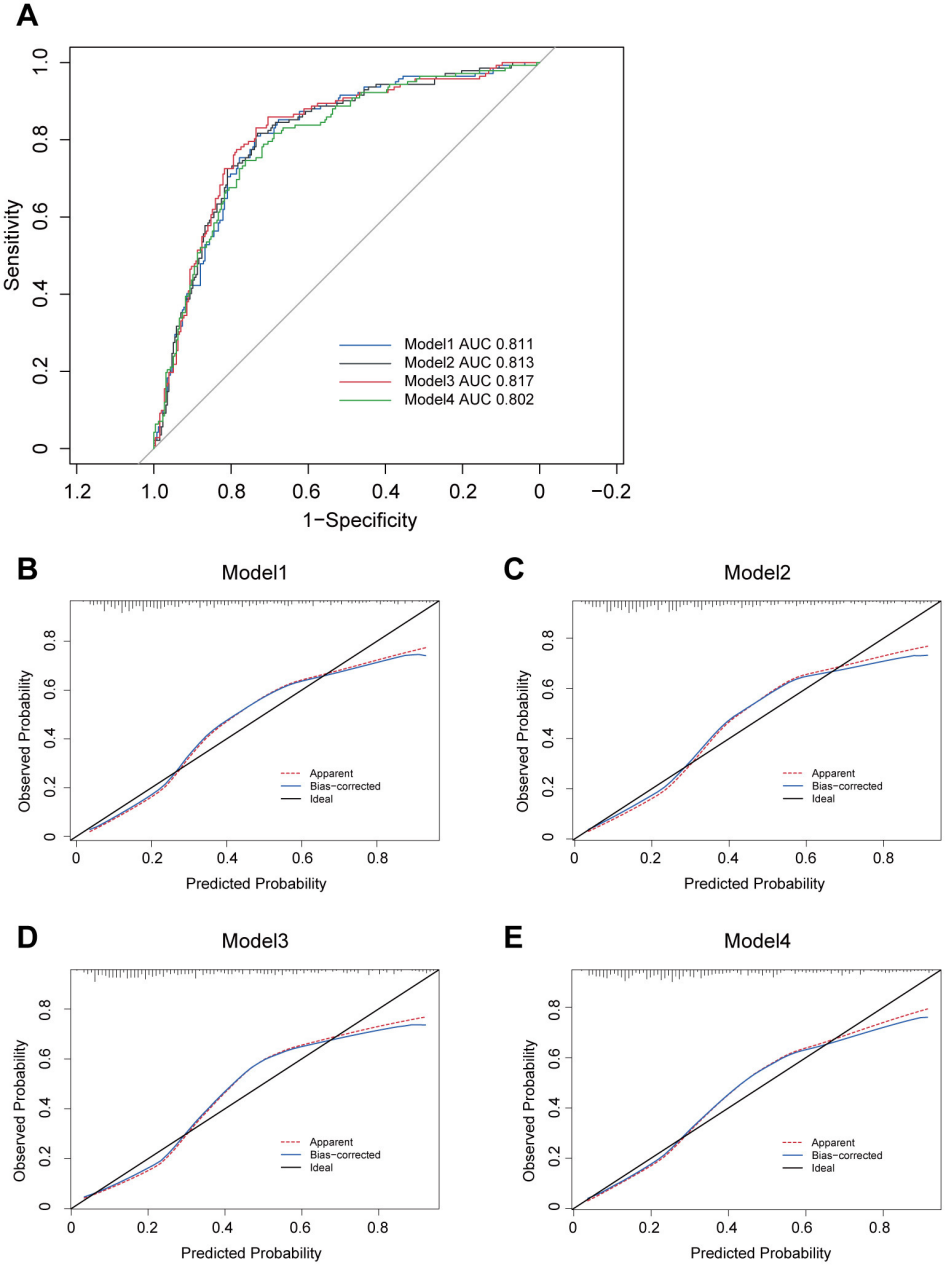
Four models for patients with kidney stones predicting post-PCNL SIRS. **(A)** ROC curves for four models including five independent risk factors predicting post-PCNL SIRS. Model 3 showed the highest AUC with 0.817. The calibration curve showed good fitting of Model 1 **(B)**, Model 2 **(C)**, Model 3 **(D)** and Model 4 **(E)**.

**Figure 3:**
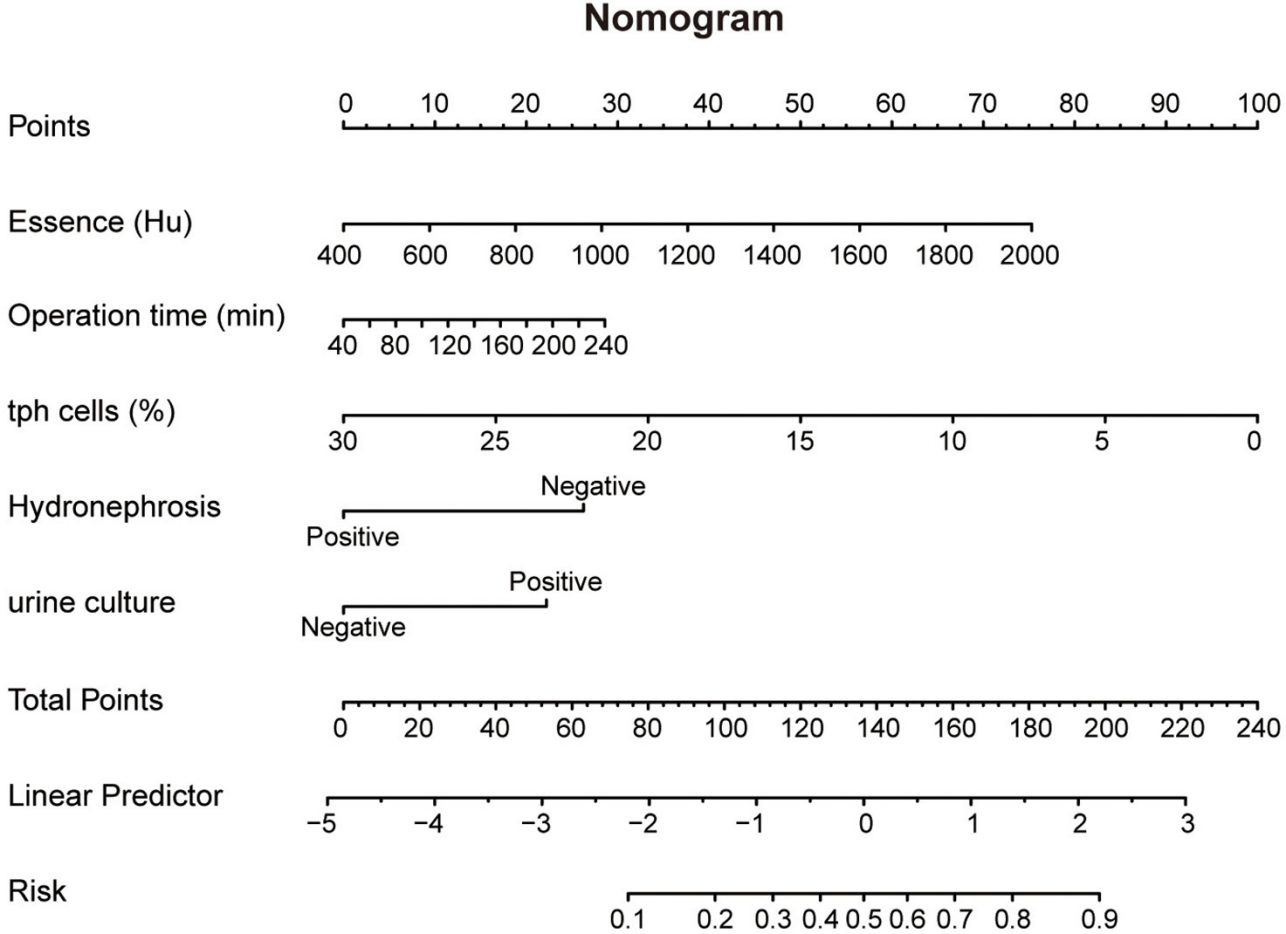
Nomogram of Model 3 predicting post-PCNL SIRS. Nomogram of Model 3 for predicting post-PCNL SIRS. Essence, operation time, percentage of Tph cells, Hydronephrosis and urine culture are marked as “points”. Total points by adding the five points can predict SIRS risk.

**Figure 4:**
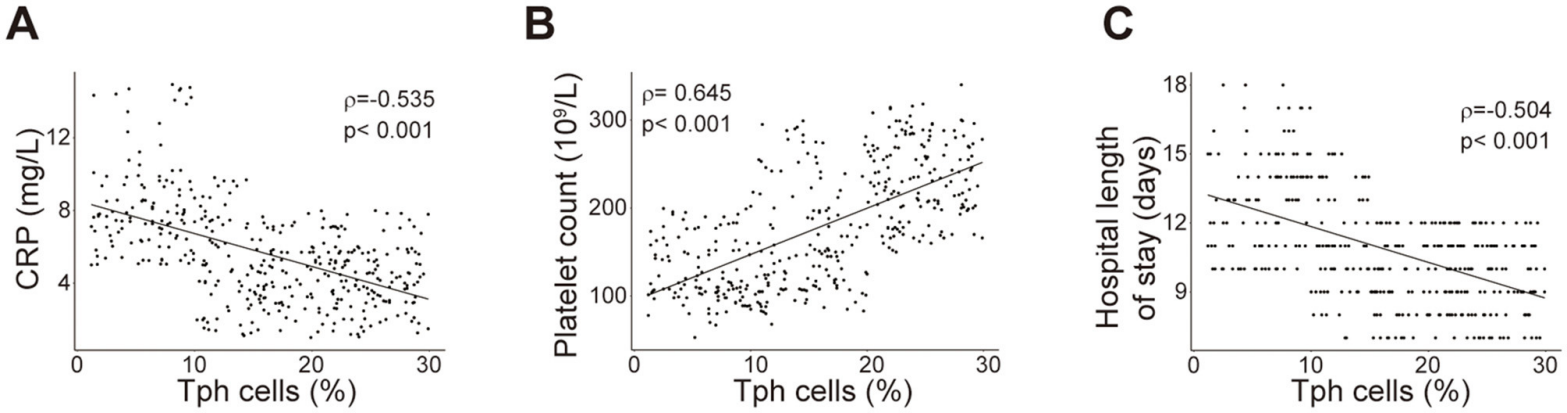
Correlation analysis between Tph cells and clinical outcomes. Correlation analyses were conducted between Tph cells and CRP **(A)**, Platelet counts **(B)**, and hospital length of stay **(C)**. Each plot represented data of one patient. Spearman's coefficient of correlation was used and r and p values for each parameter were listed.

Furthermore, ROC curve analyses were performed for these models, revealing reasonable predictive capabilities with AUC values of 0.811, 0.813, 0.817 and 0.802, respectively (Figure 2A). Model 3 exhibited the superior discriminative performance among the four (Figure 2A). The incidence rate of post-PCNL SIRS was calculated, and the calibration curves for all models demonstrated good fitting (Figures 2B–E). Predictive nomograms, based on the model 3 according to max AUC value, were generated to estimate the probability of post-PCNL SIRS occurrence (Figure 3). To visually access the relationship between Tph cell levels and clinical outcomes, we performed Spearman's correlation analysis between Tph cells and C-reactive protein (CRP) (Figure 4A), platelet counts (Figure 4B), and hospital length of stay (Figure 4C). The analysis revealed a significant negative correlation between Tph cells and CRP (r = −0.535, *p* < 0.001; Figure 4A) as well as hospital length of stay (r = −0.504, *p* < 0.001; Figure 4C). In contrast, Tph cells demonstrated a positive correlation with platelet counts (r = 0.645, *p* < 0.001; Figure 4B).

## Discussion

In this study, we pioneered the reporting of circulating Tph cells, rather than circulating Tfh cells, as an independent preoperative risk factor for post-PCNL SIRS. Through meticulous multivariate analysis, we unveiled four models grounded in circulating Tph cells, each displaying commendable discriminatory ability with AUCs exceeding 0.8. Model 1 encompassed low percentage of circulating Tph cell, high stone density, prolonged operation time, negative hydronephrosis status, and positive urine nitrite. Model 2 included similar features but with positive urine leukocytes instead of urine nitrite. Model 3 featured positive urine bacteria, and Model 4 comprised positive urine nitrite and leukocytes, all in the context of low percentage of Tph cell, high stone density, and negative hydronephrosis. Among these models, ROC curve analysis unveiled Model 3 as the superlative, with an AUC of 0.817.

For the first time, circulating Tph cells has been implicated as an independent preoperative risk factor for post-PCNL SIRS. In preceding investigations, Tph cells have been identified as playing a pivotal pathogenic role in a plethora of autoimmune diseases, including SLE (10), RA (8), IgG4-related disease (11), type 1 diabetes (12), IgA nephropathy (13), psoriasis vulgaris (14), active ulcerative colitis (15) and anti-neutrophil cytoplasmic antibody (ANCA)-associated vasculitis (AAV) (20). Our study introduces a novel frontier by uncovering the role of Tph cells in non-autoimmune conditions. We demonstrated that the low percentage of circulatory Tph cells are associated with elevated rates of post-PCNL SIRS. The potential inflammatory mechanisms may be as follows: Tph cells are multifunctional, capable of secreting a variety of cytokines, including interleukin 21 (IL-21), IL-10, interferon g (IFN-g), thereby playing a role in controlling bacterial and viral infections within the body (8, 20). Furthermore, Tph cells promote the differentiation and maturation of B cells into plasma cells (8, 20), which subsequently produce and secrete antigen-specific antibodies. This process facilitates pathogen clearance and neutralizes some of the endotoxins released from kidney stones following PCNL.

In this study, prolonged operation time was identified as an independent risk factor for post-PCNL SIRS. During the execution of PCNL, excessive renal pelvic pressure can precipitate pyelotubular, pyelolymphatic, and pyelovenous reflux, potentially facilitating the translocation of bacteria and endotoxin into systemic circulation (21). Notably, the incidence of postoperative fever markedly escalates when renal pelvic pressure attains or surpasses 20 mmHg (22). Extended operation times result in a longer duration for renal pelvic pressure to surpass a critical threshold, thereby augmenting the chances of bacteria and endotoxin entering the circulation. Furthermore, longer operative durations equate to increased surgical exposure, which can exacerbate the risk of intraoperative infections, ultimately contributing to post-PCNL SIRS.

This study introduces the novel finding that high stone density is an independent risk factor for post-PCNL SIRS. While direct evidence linking high stone density to an elevated rate of post-PCNL SIRS remains elusive, we postulate several plausible explanations: (1) Stones with high density necessitate more extensive operative time for removal, potentially enhancing the likelihood of bacteria and endotoxin entering the circulation; (2) Stones exhibiting high CT values may require greater energy for lithotripsy, which could facilitate the entry of bacteria and endotoxins into the circulation, thereby elevating the risk of post-PCNL SIRS.

In this study, we have discovered that hydronephrosis exhibits a negative correlation with the incidence of post-PCNL SIRS, despite its identification as a risk factor for infection subsequent to extracorporeal shock wave lithotripsy (SWL) (23). The influence of hydronephrosis on PCNL outcomes remains contentious. On the one hand, hydronephrosis has been implicated in lower stone-free rates following PCNL (24–26). Conversely, other studies have failed to establish any impact of hydronephrosis on stone-free rates post-PCNL (27, 28). Our investigation specially focused on the presence of hydronephrosis in PCNL patients and revealed that the absence of hydronephrosis was associated with an increased risk of post-PCNL SIRS. We hypothesize that this may stem from: (1) Patients with kidney stones without hydronephrosis experiencing a more insidious and protracted disease course; (2) the greater technical challenge and prolonged operative time associated with the puncture and stone removal procedure in such patients.

Positive urine bacteria detection has emerged as an independent risk factor for post-PCNL SIRS. Prior research has underscored the significance of preoperative urine culture, intraoperative renal pelvic urine culture, and stone culture as predicative markers for post-PCNL SIRS (29–31). Nevertheless, performing these cultures, particularly stone culture, is neither timely nor convenient. The turnaround time for all culture results exceeds 2–3 days, and the positivity rate is relatively low. Given the impracticality of obtaining stone culture prior to lithotripsy, its clinical utility is inherently limited. In contrast, urine bacterial examination offers a more expedient alternative, delivering results within 4 h, thereby enhancing its clinical applicability.

The present investigation, for the first time, demonstrates the potential of circulating Tph cells as a diagnostic biomarker for post-PCNL SIRS. A preoperative nomogram, devised using five independent risk factors predictive of post-PCNL SIRS, was formulated in this study. This tool may aid physicians in identifying high-risk patients preoperatively, enabling the adoption of more prudent strategies, such as optimizing operation time, intensifying perioperative monitoring, and preemptively administering higher-grade antibiotics. Our findings may pave the way for future research exploring the prognostic value of T lymphocyte subsets in predicting post-PCNL SIRS.

In addition to serving as a risk indicator for post-PCNL SIRS, Tph cells may also represent a potential therapeutic target to mitigate inflammatory risk during the perioperative period. Previous studies have demonstrated that cells such as ITGA5^+^ fibroblasts can induce the differentiation of Tph cells through TGF-b1 signaling (32, 33). Additionally, *in vitro* studies have shown that IL-2 stimulation reduces Tph cell levels, suggesting that neutralizing IL-2 may promote the differentiation of Tph cells (34). These findings indicate that modulating inflammatory cytokines, such as TGF-b1 and IL-2, could enhance Tph cell differentiation. This, in turn, supports the differentiation of B cells into plasma cells, which secrete a range of antigen-specific antibodies. This process aids in pathogen clearance and neutralizes endotoxins derived from kidney stones following PCNL. Although further clinical studies are required to assess the efficacy and safety of therapeutic strategies targeting Tph cells, this approach holds significant promise and potential.

Despite these strengths, our study is not without limitations. Firstly, while five independent risk factors for post-PCNL SIRS were identified in Model 3, the robustness of this model necessitates validation by independent researchers. Secondly, the precise mechanisms underlying the observed association between post-PCNL SIRS and circulating Tph cells remain elusive, necessitating further investigation, particularly at the molecular level. Thirdly, although procalcitonin and C-reactive protein are established biomarkers for predicting post-PCNL SIRS (16), their preoperative assessment was not routine at our institutions, hence their exclusion from this study. Lastly, potential selection bias is inherent in retrospective studies conducted at limited centers, and our study is no exception. Comprehensive, multicenter trials involving larger cohorts are warranted to elucidate the clinical implications of the proposed nomogram based on circulating Tph cells.

## Conclusion

This study represents the first report of circulating Tph cells as an independent risk factor for post-PCNL SIRS. A novel nomogram, grounded in the analysis of this T cell subset, has been devised to predict the occurrence of post-PCNL SIRS. The findings of this research may empower clinicians to identify kidney stone patients at high risk of developing SIRS, thereby facilitating timely and targeted therapeutic interventions.

## Data Availability

The original contributions presented in the study are included in the article/Supplementary material, further inquiries can be directed to the corresponding authors.
